# Lung Separation in the Morbidly Obese Patient

**DOI:** 10.1155/2012/207598

**Published:** 2012-02-06

**Authors:** Javier H. Campos, Kenichi Ueda

**Affiliations:** Department of Anesthesia, University of Iowa Healthcare, Iowa City, IA 52242, USA

## Abstract

Lung separation techniques in the morbidly obese patient undergoing thoracic or esophageal surgery may be at risk of complications during airway management. Access to the airway in the obese patient can be a challenge because they have altered airway anatomy, including a short and redundant neck, limited neck extension and accumulation of fat deposition in the pharyngeal wall contributing to difficult laryngoscopy. Securing the airway is the first priority in these patients followed by appropriate techniques for lung separation with the use of a single-lumen endotracheal tube and a bronchial blocker or another alternative is with the use of a double-lumen endotracheal tube. This review is focused on the use of lung isolation devices in the obese patient. The recommendations are based upon scientific evidence, case reports or personal experience. Fiberoptic bronchoscopy must be used to place and confirm proper placement of a single-lumen endotracheal tube, bronchial blocker or double-lumen endotracheal tube.

## 1. Introduction

Lung separation techniques are designed to facilitate surgical exposure while providing one-lung ventilation (OLV) in patients undergoing thoracic or esophageal surgery. This is achieved by the use of a double-lumen endotracheal tube (DLT) or bronchial blockers such as the Arndt wire-guided blocker, the Cohen endobronchial blocker, Fuji Uniblocker, the Univent tube, or the EZ Blocker [1, 2]. The effectiveness of lung separation, lung collapse, and clinical performance has been evaluated in patients with average or above-normal weight among DLTs or bronchial blockers with similar results [3, 4]. In contrast, there is only one study involving lung separation techniques with the use of a single-lumen endotracheal tube and a bronchial blocker (Arndt blocker) compared with a left-sided DLT in morbidly obese patients requiring thoracic or esophageal surgery [5]. The prevalence of morbid obesity is increasing in the world; in fact, one-third of the adult population in the United States is obese, and it is estimated that between 5% and 10% are considered severely or morbidly obese. An obese person is one with a body mass index (BMI) >30 kg/m^2^ and morbidly obese when the BMI >35 kg/m^2^ [6].

 Management of the airway in the obese patient can be a challenge because they have altered airway anatomy, including a short and large neck, limited neck extension, and accumulation of fat deposition in the pharyngeal wall contributing to difficult laryngoscopy [7, 8]. The obese patient often has increased residual gastric volumes and increases acidity of gastric fluid, making them more vulnerable to aspiration [9, 10]. Intubation with a DLT can be more difficult than intubation with a single-lumen endotracheal tube because the DLT is larger in circumferential diameter than a single-lumen endotracheal tube, also the lack of a bevel at the tip of the DLT can make an otherwise acceptable view of the glottis obscured [11]. Some of these obese patients also have obstructive sleep apnea (OSA) [12–14].

Changes in pulmonary mechanics, limited reserve, and circulation in morbidly obese patients can make them prone to desaturation [15–17]. The purpose of this review will be focused on: (1) airway implications in the obese patient and (2) the use of bronchial blockers and DLTs during lung separation techniques in the obese patient.

## 2. Airway Implications in the Obese Patient Requiring Lung Separation

Morbidly obese patients present with abundant fatty tissue on the neck, thoracic wall, and abdomen. The pharynx and larynx have excess fatty tissue; therefore, these patients are known to develop airway obstruction by the abundance of soft tissue in the upper airway. During intubation this excess of fatty tissue may increase the risk of access and patency of the airway [7]. Also, many of adult obese patients have OSA [12]. All of these comorbidities will contribute to difficulties managing their airways.

However, not all obese patients will present problems during intubation when a single-lumen endotracheal tube is used. Ezri et al. [18] reported that the BMI per se has no influence on the difficulty of laryngoscopy. Meyer [19] also has shown that difficult intubation in morbidly obese patients was similar to that of nonobese patients. Increase in neck circumference is a leading risk factor for difficult intubation [20]. A study by Brodsky et al. [8] involving 100 consecutive morbidly obese patients requiring tracheal intubation with a single-lumen endotracheal tube showed that neck circumference was the best single predictor of problematic intubation; however, neither absolute obesity nor increased BMI was associated with difficult intubation. In addition, in the obese patient a disproportionately large base of the tongue is considered a predisposing factor for difficult laryngoscopy [21].

The best preoperative predictor of potential difficulty with tracheal intubation in a morbidly obese patient is a Mallampati score of III or IV [8, 22]. Once the airway is recognized as being potentially difficult, a careful examination of the patient ensues to determine an optimal technique to secure the airway.

## 3. Use of Bronchial Blockers during Lung Separation in the Obese Patient

In patients who require one-lung ventilation (OLV) and present with the dilemma of a difficult airway, such as the morbidly obese patient, the primary goal after appropriate airway anesthesia is achieved, is to establish an airway with a single-lumen endotracheal tube placed orally with the aid of a flexible fiberoptic bronchoscope while the patient is awake [23]. In select patients who seem easy to ventilate by a mask, intubation can be performed with an Airtraq laryngoscope or with a video laryngoscope [24]. Video laryngoscopy clearly can allow visualization of the pharynx, epiglottis, and vocal cords, plus visualization of the passage of the single-lumen endotracheal tube after induction of anesthesia.

An alternative when securing the airway prior to placing a lung separation device is the use of a laryngeal mask airway with the aid of a flexible fiberoptic bronchoscope; single-lumen endotracheal tube can be passed through the laryngeal mask airway. When using a large size laryngeal mask airway and passage of a single-lumen endotracheal tube size 6.5 mm ID is recommended. The use of the laryngeal mask airway C Trach has been reported to be an efficient airway device for ventilation and tracheal intubation in case of a difficult airway in morbidly obese patients [25]. However, the potential for airway trauma may be higher with this exchange technique.

**Figure 1:**
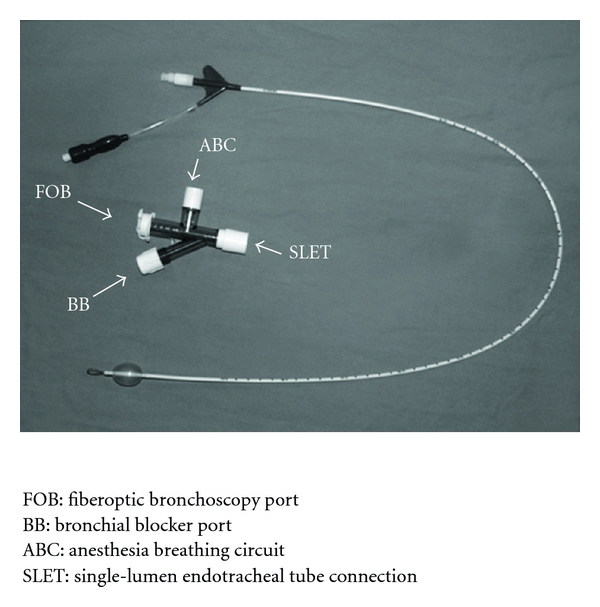
displays the Arndt blocker.

**Figure 2:**
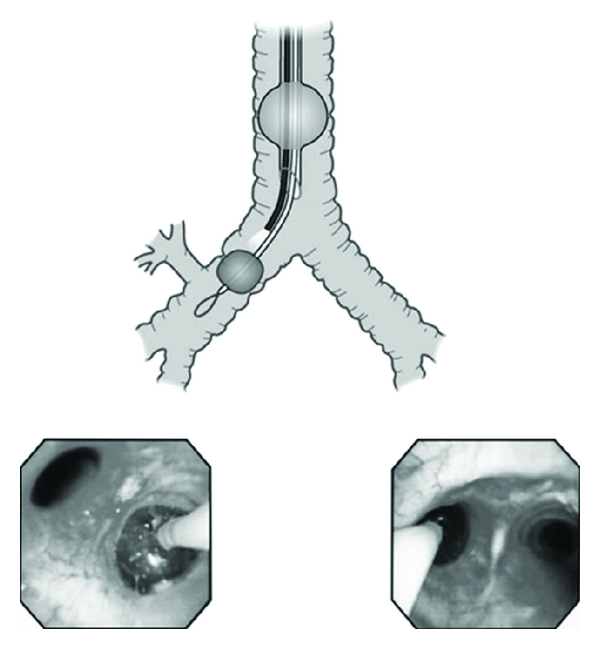
The proper position of a bronchial blocker seen with fiberoptic bronchoscopy.

 Bronchial blockers can be used following anesthesia induction and after the airway has been secured with a single-lumen endotracheal tube. The common bronchial blockers used through a single-lumen endotracheal tube include the Arndt blocker (adult sized 7.0 and 9.0 F), the Cohen endobronchial blocker (size 9.0 F), the Fuji Uniblocker (size 9.0 F) [4], the Univent tube, or the EZ blocker [26].  Figure 1 displays the Arndt blocker. One advantage of one-time intubation with a single-lumen endotracheal tube is that it allows for the conversion to OLV with insertion of the bronchial blocker and simple removal of this at the end of a procedure if postoperative ventilatory support is needed [27]. When a bronchial blocker is used, specifically size 9.0 F, the smallest acceptable single-lumen endotracheal tube size recommended is 8.0 mm ID. However, if a smaller single-lumen tube is used then a 7 F Arndt blocker is recommended. It is important to have enough space between the bronchial blocker and the flexible fiberoptic bronchoscope so navigation can be achieved with the single-lumen endotracheal tube. To achieve OLV the bronchial blocker must be advanced to the bronchus where lung collapse is required. Once the blocker is within the bronchus and the patient is turned into the lateral decubitus position, the inflation of the endobronchial balloon should be done under direct vision with the aid of a flexible fiberoptic bronchoscope while the lung is not ventilated. The amount of air needed to achieve a complete seal within the bronchus in adults ranges between 5 and 8 mL of air. The optimal position of a bronchial blocker in the left bronchus is when the blocker's balloon outer surface is seen at least 10 mm below tracheal carina inside the blocked bronchus. For the use of a right-side bronchus the depth on insertion of the blocker and balloon will depend upon the anatomical distance between the tracheal carina and the orifice of the right upper lobe bronchus. The optimal positions for all bronchial blockers should be confirmed with a flexible fiberoptic bronchoscope [28].  Figure 2 displays the proper position of a bronchial blocker seen with fiberoptic bronchoscopy. It is our personal experience and opinion that in the obese patient in order to expedite lung collapse the center channel of the bronchial blocker should be attached to wall suction for a few minutes, this maneuver will facilitate lung collapse. After OLV is completed and the surgical procedure has ended, if postoperative mechanical ventilation is needed, withdrawal of the bronchial blocker ensues, leaving the single-lumen endotracheal tube in place.

A common problem with the use of the bronchial blockers is that malpositions occur more often than with the DLTs [4]. Potential complications related to the use of the bronchial blocker might include inclusion of a bronchial blocker or the nylon guide wire into the stapling line [29, 30]. This is why communication with the surgical team regarding the placement of a bronchial blocker in the surgical side is crucial. Removal of the guide wire in the Arndt blocker is mandatory prior to establishment of OLV.  Table 1 displays the characteristics of the bronchial blockers.

## 4. Use of a Double-Lumen Endotracheal Tube in the Obese Patient

The obese patient is at risk for difficulties placing a DLT. For the vast majority of cases that required lung separation, a left-sided DLT will be more commonly used because of its greater margin of safety when compared to the right-sided DLT. Another important consideration while managing the airway in the obese patient is that many of the obese patients also have OSA because of adipose deposition around the pharynx, which collapses the airway and increases the chance of failure mask ventilation [31]. Moreover, obese patients have limited oxygen reserve due to a decreased functional residual lung volume [32]. More recently, a video-assisted tracheal intubation device for single-lumen endotracheal tube placement showed significant improvement of intubation time and reduction of hypoxic event during induction in morbidly obese patients [24]. In practice there are three different ways to place a left-sided DLT in a patient with a difficult airway. The first involves the use of airway topical anesthesia and awake fiberoptic bronchoscopy with passage of the flexible fiberoptic bronchoscope through the bronchial lumen of the DLT, where the tube is advanced under bronchoscope guidance. The second technique involves the use of ancillary lighted devices or video laryngoscopes that increase the visualization field of the epiglottis, vocal cords, and passage of the tube. A malleable, lighted stylet has been reported; by using the device within the endobronchial lumen of the DLT, where the tip of the bulb was positioned distally at the tip of the DLT in patients with difficult airways. Others have reported the use of a fiberoptic laryngoscope, the WuScope during placement of a DLT in patients with abnormal airway anatomy. One of the advantages of the fiberoptic laryngoscope is that it protects against rupture of the endotracheal cuff during laryngoscopy because the DLT is enclosed with the laryngoscope blade. Disadvantage of this device include the need for smaller sizes of DLTs, such as 35–37 F [23].

The GlideScope video laryngoscope has been used in patients with a difficult airway during placement of a DLT. Another alternative is to intubate the patient's trachea with a single-lumen endotracheal tube during an awake fiberoptic bronchoscopy or after induction of anesthesia, and then a tube exchange technique can be used to replace the existing tube for a DLT after general anesthesia is induced. For a tube exchange catheter to function, it must have a hollow center channel and universal adapters to insufflate oxygen. The exchange catheter must have a flexible tip distally to avoid airway lacerations, be long in length, and have outer markings to control the depth of insertion while in use. For a DLT, the exchange catheter should be at least 83 cm long. The airway aintree tube exchanger has a large internal diameter that allows fiberoptic bronchoscopy guidance. Also, a 14 F exchange catheter can be used to facilitate insertion of 39 and 41 F DLTs. For a 35 or 37 F DLT, a single or double airway exchange catheter can be used.

The airway exchange catheter, single-lumen endotracheal tube, and the DLT combination should be tested in vitro before the exchange. A sniffing position will facilitate tube exchange. After the airway exchange catheter is lubricated, it is advanced through a single-lumen endotracheal tube. The airway catheter should not be inserted deeper than 24 cm from the lips to avoid accidental rupture or laceration of the trachea or bronchi. The balloon of the single-lumen endotracheal tube is deflated and then maintaining the airway exchange catheter at approximately 24 cm from the lips in a 170 cm tall subject, the single-lumen endotracheal tube is removed and the DLT is advanced through endobronchial lumen; a laryngoscope should be used to facilitate the guidance of the DLT.

## 5. Comparison of a Bronchial Blocker and a DLT in Obese Patients

A recent study comparing the use of a single-lumen endotracheal tube with a left-sided DLT involving morbidly obese patients requiring lung separation showed that the first attempt success during laryngoscopy of DLT placement was no different than with the degree of difficulties to that of a single-lumen endotracheal tube placement and with Arndt blocker in morbidly obese patients [5]. In both groups 2/25 and 3/25 of the patients studied, respectively, required a second or third attempt at intubation while placing DLTs or single-lumen endotracheal tubes. Also, in the DLT a tube exchanger technique was required in two of the patients studied. However, after the bronchial blockers or DLTs were in place there was no clinical difference in terms of lung collapse; in both groups the lung collapse was rated as excellent or good and only 1 patient in each group had poor lung collapse. This study clearly showed no difference between bronchial blockers and DLTs in this patient population.

## 6. Confirmation of Placement of a Left-Sided DLT

**Figure 3:**
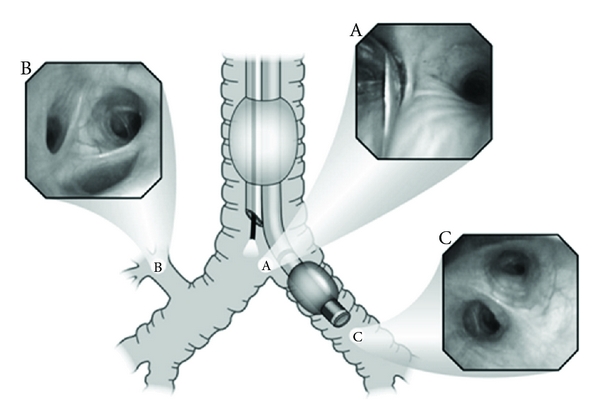
The proper position of a left-sided DLT when seen with a fiberoptic bronchoscope. (A) View of the tracheal carina, towards the left side the endobronchial lumen is visible, the left main bronchus with the outer surface of the endobronchial balloon is seen below tracheal carina. (B) View of the 3 orifices of the right upper lobe bronchus (apical, anterior, and posterior segments). (C) Clear view of the left upper and left lower bronchus.

After the DLT is advanced in the patient's trachea and confirmation of end tidal CO_2_ with capnometry is obtained the optimal position of the DLT is achieved with a flexible fiberoptic bronchoscope. For a left-sided DLT the optimal position is defined when the fiberoptic bronchoscope is passed through the tracheal lumen and an unobstructed view of the right main bronchus is seen with a clear view of the tracheal carina. It is important to distinguish the right upper lobe bronchus and the 3 branches which are apical, anterior, and posterior segments of the right upper lobe. This is the only structure that has 3 orifices. To the left (left bronchus) there is an endobronchial lumen, and the edge of the fully inflated balloon is seen approximately 10 mm below tracheal carina in the left bronchus. Then the fiberoptic bronchoscope is readvanced through the endobronchial lumen where the distal tip of the tube is seen and an unobstructed view of the left upper and left lower lobe bronchus is seen. This view should be obtained in supine and then lateral decubitus position [28].  Figure 3 shows a left-sided DLT. Potential complications with the use of DLTs include airway trauma and trachea or bronchial ruptures; reportedly more common with the use of smaller DLTs (i.e., 35 F) [1].

## 7. Summary

Obese patients requiring lung separation devices may be at risk of complications during airway management. A key element during the preoperative assessment is recognition and identification of the potentially difficult airway. Then the safest way to establish an airway is by securing the airway with a single-lumen endotracheal tube placed orally with the aid of flexible fiberoptic bronchoscopy. Once the airway is secured a bronchial blocker can be used to facilitate lung separation. A different alternative can be the use of a DLT with a tube exchanger technique assisted with a video laryngoscope. Fiberoptic bronchoscopy must be used to place and confirm proper placement of single-lumen endotracheal tube, bronchial blockers or DLTs. Because of the limited research available on obesity and lung separation techniques, further studies are needed to determine the best device or best technique to achieve surgical exposure and lung collapse in this patient population.

## Figures and Tables

**Table 1:** Characteristics of the bronchial blockers.

	Cohen blocker	Arndt blocker	Fuji uniblocker	EZ blocker
Size	9 F	5 F, 7 F, and 9 F	9 F	7 F
Balloon shape	Spherical	Spherical or elliptical	Spherical	Spherical
Guidance mechanism	Wheel device	Nylon wire loop	None, preshaped tip	None
Smallest recommended *SLET for coaxial use	9 F—8.0 SLET	5 F—4.5 SLET 7 F—7.0 SLET 9 F—8.0 SLET	9 F—8.0 SLET	7.5
Murphy eye	Present	Present in 9 F	Not present	No
Center channel	1.6 mm I.D.	1.4 mm I.D.	2.0 mm I.D.	1.4 mm I.D.

*SLET = Single-lumen endotracheal tube.
